# Screen-Printed Carbon Electrodes with Macroporous Copper Film for Enhanced Amperometric Sensing of Saccharides

**DOI:** 10.3390/s22093466

**Published:** 2022-05-02

**Authors:** Radovan Metelka, Pavlína Vlasáková, Sylwia Smarzewska, Dariusz Guziejewski, Milan Vlček, Milan Sýs

**Affiliations:** 1 Department of Analytical Chemistry, Faculty of Chemical Technology, University of Pardubice, Studentská 573, 532 10 Pardubice, Czech Republic; radovan.metelka@upce.cz (R.M.); pavlina.vlasakova@student.upce.cz (P.V.); 2 Department of Inorganic and Analytical Chemistry, Faculty of Chemistry, University of Lodz, 12 Tamka Str., 91-403 Lodz, Poland; sylwia.smarzewska@chemia.uni.lodz.pl (S.S.); dguziejewski@uni.lodz.pl (D.G.); 3 Joint Laboratory of Solid State Chemistry, Faculty of Chemical Technology, University of Pardubice, Studentská 84, 532 10 Pardubice, Czech Republic; milan.vlcek@upce.cz

**Keywords:** non-enzymatic sensors, colloidal crystal templating, porous copper electrodes, amperometric detection, sensing of saccharides

## Abstract

A porous layer of copper was formed on the surface of screen-printed carbon electrodes via the colloidal crystal templating technique. An aqueous suspension of monodisperse polystyrene spheres of 500 nm particle diameter was drop-casted on the carbon tracks printed on the substrate made of alumina ceramic. After evaporation, the electrode was carefully dipped in copper plating solution for a certain time to achieve a sufficient penetration of solution within the polystyrene spheres. The metal was then electrodeposited galvanostatically over the self-assembled colloidal crystal. Finally, the polystyrene template was dissolved in toluene to expose the porous structure of copper deposit. The morphology of porous structures was investigated using scanning electron microscopy. Electroanalytical properties of porous copper film electrodes were evaluated in amperometric detection of selected saccharides, namely glucose, fructose, sucrose, and galactose. Using hydrodynamic amperometry in stirred alkaline solution, a current response at +0.6 V vs. Ag/AgCl was recorded after addition of the selected saccharide. These saccharides could be quantified in two linear ranges (0.2–1.0 μmol L^−1^ and 4.0–100 μmol L^−1^) with detection limits of 0.1 μmol L^−1^ glucose, 0.03 μmol L^−1^ fructose, and 0.05 μmol L^−1^ sucrose or galactose. In addition, analytical performance of porous copper electrodes was ascertained and compared to that of copper film screen-printed carbon electrodes, prepared ex-situ by the galvanostatic deposition of metal in the plating solution. After calculating the current densities with respect to the geometric area of working electrodes, the porous electrodes exhibited much higher sensitivity to changes in concentration of analytes, presumably due to the larger surface of the porous copper deposit. In the future, they could be incorporated in detectors of flow injection systems due to their long-term mechanical stability.

## 1. Introduction

Nowadays, non-enzymatic glucose sensors (NEGSs) are of increasing interest to the scientific community, unlike traditional sensors employing an enzyme layer containing a glucose oxidase due to the possible decrease in catalytic activity of the enzyme arising from the immobilization and low guarantee of long-term stability [1]. However, it is necessary to remember that traditional enzymatic sensors are usually more selective than non-enzymatic ones due to the very specific catalytic pathway of the natural origin catalyst [2]. In addition to clinical analysis, where the determination of glucose level in the blood predominates, non-enzymatic sensors can serve as useful analytical tools in agriculture for control of crop maturity [3], in food technology for bio-fermentation process monitoring [4], and in food safety control to detect lactose in lactose-free milk dairy products intended for the lactose-intolerant population [5].

Non-enzymatic sensors designed to determine simple carbohydrates operate predominantly in the amperometric mode, where glucose represents the most common target analyte. Because glucose, galactose, and fructose are the end products of carbohydrate digestion and only glucose enters the bloodstream, NEGSs have to be selective for common interferences, such as ascorbic acid, dopamine, acetaminophen, and uric acid [6]. Unlike the previous case, foodstuffs can be considered much more complex samples because they can contain a variety of simple monosaccharides and disaccharides, which are readily electrochemically oxidized to the corresponding carboxylic acid in an alkaline environment [7]. Unfortunately, only a few of the NEGSs developed to date are selective to glucose in the presence of other saccharides [8,9,10].

Generally, the electrocatalytic process for glucose oxidation to gluconolactone, which is subsequently hydrolyzed to gluconic acid, is usually mediated by the Me(II)/Me(III) redox couple in the alkaline solution, where Me(III) species acted as an electron transfer mediator [11]. Nanostructured D-block metals, such as copper [12], nickel [13], and iron [14], predominate in the development of glucose non-enzymatic sensors. Moreover, Co(III)/Co(IV) [15] and Mn(III)/Mn(IV) redox couples [16] have been used sporadically.

To increase the sensitivity of amperometric non-enzymatic sensors, different approaches have been used for the three-dimensional extent of active surface area. Several approaches were utilized for this purpose, namely nanomaterials decorated by island structures of electrochemically deposited metal films [17,18], fabricated nanocomposites of polymers and metal oxides [19,20], porous metallic materials [21,22,23,24,25], or their combinations [26].

In this paper, a colloidal crystal templating technique [27] has been used for the preparation of porous copper layers onto surfaces of screen-printed carbon electrodes (SPCEs) which were fabricated by the screen printing of commercially available carbon ink on alumina ceramic supports. Detailed morphological characterization of obtained porous structures was performed using scanning electron microscopy. Electroanalytical properties of the porous copper film electrodes (pCuFEs) were investigated using cyclic voltammetry and amperometry of selected saccharides, namely glucose, sucrose, fructose, and galactose.

The results showed that the developed non-enzymatic porous copper sensors (NEPCSs) are selective to glucose in the presence of common electroactive interferences (ascorbic acid and uric acid) and show enhanced current response compared to non-porous copper film electrodes, allowing their feasible application in clinical and food analysis. However, it was also found that NEPCSs provide an overall current response for all saccharides tested. Therefore, a pre-separation step is necessary if quantification of individual saccharides is required in terms of their application in food and environmental analysis, where they could serve as sensitive electrochemical detection systems [28,29,30,31].

## 2. Materials and Methods

### 2.1. Chemicals

D-glucose (p.a), D-(−)-fructose (≥99%), sucrose (p.a), D-(+)-galactose (98%), copper(II) nitrate trihydrate (p.a.), ascorbic acid (p.a.), uric acid (≥99%), and suspensions of monodisperse polystyrene beads with particle sizes of 200 or 500 nm were purchased from Sigma Aldrich (Prague, Czech Republic). Other chemicals, such as toluene (p.a.), nitric acid (65%), sodium hydroxide (≥97.0%), and acetone (≥99.0%) were obtained from Lach-Ner s.r.o (Neratovice, Czech Republic). All aqueous solutions were prepared using deionized water with resistivity higher than 18.3 MΩ cm, which was obtained from the Millipore Milli-Q^®^ system from Merck (Darmstadt, Germany).

### 2.2. Apparatus

Electrochemical measurements were carried out in a voltammetric glass cell with a conventional three-electrode configuration with the use of a screen-printed carbon electrode (SPCE) plated with a porous copper layer (pCuFE) (working), a silver chloride electrode (length 12.5 cm) with 3.0 mol L^−1^ KCl salt bridge (reference) from Metrohm Česká republika s.r.o. (Prague, Czech Republic), and Pt sheet auxiliary electrode from Elektrochemické detektory, s.r.o. (Turnov, Czech Republic). The abovementioned electrodes were connected to a potentiostat/galvanostat AUTOLAB PGSTAT101 from Metrohm Česká republika s.r.o. (Prague, Czech Republic) which was operated by the NOVA 1.11 software. Images of electrode surface structures were obtained by scanning electron microscope (SEM) JEOL JSM7500F (Tokyo, Japan) using a secondary electron detector and accelerating voltage of 15 kV or 20 kV.

### 2.3. Preparation of Screen-Printed Carbon Electrodes

Bare screen-printed carbon electrodes were prepared by screen-printing of commercial carbon ink (type C10903D14) purchased from Gwent Electronic Materials Ltd. (Pontypool, UK) onto the ceramic supports (10 × 40 mm). The printing itself was done using a semi-automatic screen-printer UL 1505 A from Tesla (Prague, Czech Republic) through an etched stencil (thickness 100 µm, electrode printing area 105 mm^2^). The freshly printed electrodes were dried at 60 °C for 30 min. Furthermore, they were covered with a thin layer of PVC insulator to define a rectangular shape of the working electrode area (5 × 3 mm) while the other side was fitted with adhesive copper tape for better electrical contact.

### 2.4. Preparation of Copper Film Electrodes

To confirm an improvement of non-enzymatic amperometric sensing of saccharides using pCuFEs, it was important to make a comparison with conventional copper film electrodes (CuFEs). A rectangular shape with the size of working surface area (5 × 3 mm) was defined using a PVC insulator before the copper electrodeposition. Conventional CuFEs were prepared by galvanostatic electroplating from solution 0.1 mol L^−1^ Cu(II) in 0.01 mol L^−1^ HNO_3_ (pH 2) at −1.5 mA for 90 s. After rinsing with a stream of deionized water, the resulting CuFEs were ready for subsequent electrochemical measurements.

### 2.5. Preparation of pCuFEs

Circular plastic molds (rings) with an internal surface area of 10 mm^2^ were applied onto the working surfaces of bare SPCEs. A volume of 30 μL of monodisperse polystyrene beads suspension (0.3, 0.5, 0.7, or 1.0% *w*/*w*) with a 200 or 500 nm diameter was pipetted into such prepared molds. After evaporating deionized water for two days in a steady environment, the plastic molds were removed. The self-assembled colloidal crystals were used as templates for preparing the porous copper layers. Prior to the electrodeposition of copper film, it was necessary to insert an SPCE with a colloidal crystal template into a copper deposition solution containing the 0.1 mol L^−1^ Cu(II) in 0.01 mol L^−1^ HNO_3_ (pH 2) for at least 15 min (infiltration time). The copper plating was then carried out galvanostatically at a constant current of −1.5 mA for different deposition times, namely 30, 60, and 90 s. After copper electroplating, the electrode was rinsed with a stream of deionized water and inserted into pure toluene to dissolve the polystyrene template for 15 min. Finally, the resulting pCuFE was rinsed again with water and, after drying, used for subsequent SEM characterization and electrochemical measurements.

### 2.6. Methods

To study the electrochemical behavior of the saccharides of interest (glucose, fructose, sucrose, and galactose), a model solution with 1.0 mmol L^−1^ of each saccharide was measured using cyclic voltammetry (CV) within a potential range from 0.0 to +0.8 V vs. Ag/AgCl at the bare SPCE, CuFE, and pCuFE and in non-deaerated 0.1 mol L^−1^ NaOH of pH 13 at a potential step of 5.0 mV and scan rate of 50 mV s^−1^. Hydrodynamic amperometric measurements in the batch configuration were performed in 0.1 mol L^−1^ NaOH of pH 13 at a detection potential of +0.6 V vs. Ag/AgCl and a stirring speed of 600 rpm.

## 3. Results and Discussion

### 3.1. Preparation of pCuFEs

**Scheme 1:**
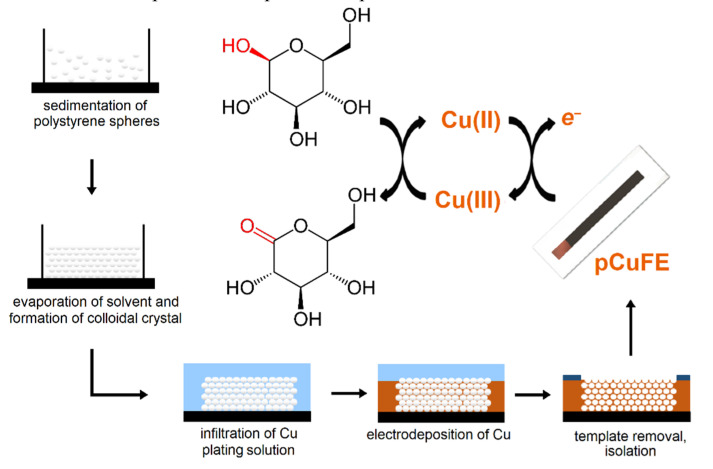
Individual steps of pCuFE fabrication with principle of glucose non-enzymatic sensing.

Porous copper films were formed using galvanostatic electrodeposition over the colloidal crystal template, made of monodisperse polystyrene spheres. The self-assembled colloidal crystal template was successfully formed on the surface of the screen-printed carbon working electrode. To properly develop the copper three-dimensional porous structure on the surface of the working electrode, several experimental parameters related to the formation of the template and electrodeposition of copper had to be optimized. After the electrodeposition, the electrode was immersed in toluene for 15 min, which readily dissolved the template and exposed the porous structure, as shown in Scheme 1.

#### 3.1.1. Colloidal Crystal Templating

Two diameters of polystyrene spheres, namely 200 nm and 500 nm, and different amounts of the particles from 0.3% to 1% *w*/*w* in the water suspension, drop-casted on the surface of the working electrode, were tested. Both types of spheres formed colloidal crystals, but in the case of lower amounts and smaller particles, the resulting porous copper film did not cover fully the surface of the electrode and consisted of isolated porous islands, presumably because it did not completely fill the rough screen-printed carbon layer. The porous copper coating was often cracked overall on the surface, and an overgrown deposit was visible even after short times of electrodeposition. On the contrary, colloidal crystal templating with larger particles and higher amounts showed a regular porous structure, which also followed the relief of the printed carbon layer and covered compactly and with only small irregularities on the surface of the working electrode. However, the use of 1% suspension often resulted in crystallite and overgrown copper deposits. Therefore, colloidal crystal templating with 0.5% suspension of 500 nm polystyrene spheres in deionized water was used as an optimum for further experiments.

#### 3.1.2. Electrodeposition of Copper Film

**Figure 1:**
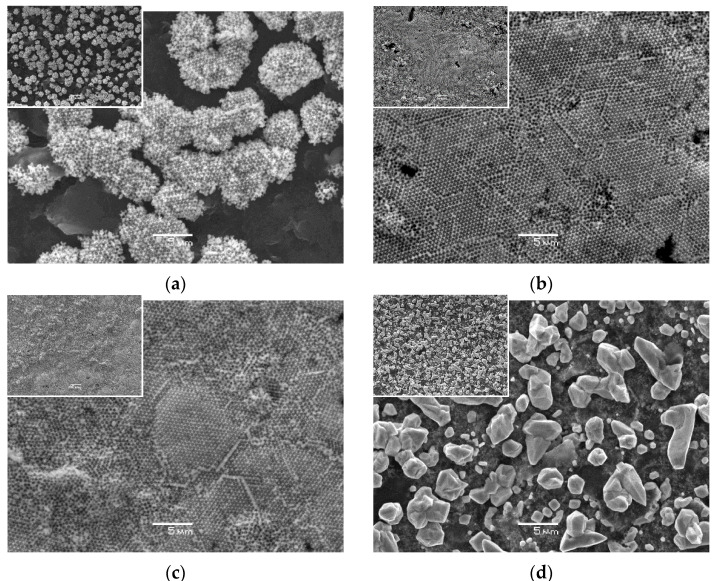
SEM images of pCuFEs prepared using colloidal crystal templating with 500 nm polystyrene spheres, constant current of –1.5 mA, and various times of copper electrodeposition from 0.1 mol L^−1^ Cu(II) in 0.01 mol L^−1^ HNO_3_ (pH 2): (**a**) 30 s; (**b**) 60 s; (**c**) 90 s; (**d**) non-porous Cu film formed without colloidal crystal template after 90 s electrodeposition for comparison (magnification 3000×, scale bar 5 µm). Insets are a lower magnification (1000×, scale bar 10 µm) of corresponding electrode surfaces.

Electrodeposition of copper using constant current provided a regular and firm metal deposit, contrary to the electrodeposition using constant potential, and was selected for further experiments. Constant currents were varied within the range from −0.5 mA to −2.0 mA. Lower currents yield weak and incomplete coverage of the electrode surface with the copper deposit, whereas higher currents led to the formation of a high amount of copper crystallites. The value of −1.5 mA provided dense coverage of carbon substrate with the copper deposit, and hence it was chosen as an optimum. Subsequently, various periods for infiltration of the plating solution into the colloidal crystal and electrodeposition of copper were tested to obtain a porous metal film on the electrode surface as homogeneously as possible. Shorter infiltration times caused the collapse of the porous structure after the copper electrodeposition and dissolution of the template in toluene. Therefore, the infiltration time of 15 min prior to the electrodeposition was found necessary for the proper development of the porous copper film. Finally, different times from 30 s to 90 s were used for ex-situ copper electrodeposition to prepare a porous copper layer. A low deposition time of 30 s resulted in partial coverage of the electrode surface with copper and consisted mainly of isolated islands of porous metal deposits (Figure 1a), similarly to the case of porous bismuth film SPCE [32]. Periods from 60 s and above ensured almost full coverage of the surface (Figure 1b), which became complete after 90 s when a solid porous copper deposit was obtained (Figure 1c). The optimized procedure allows the preparation of porous copper coatings with full coverage even on rough surfaces in the same manner as colloidal crystal templating on smooth substrates, such as gold [33,34], albeit with limited control of the thickness of the final porous layer.

### 3.2. Electrochemical Characterization of Porous CuFE

**Figure 2:**
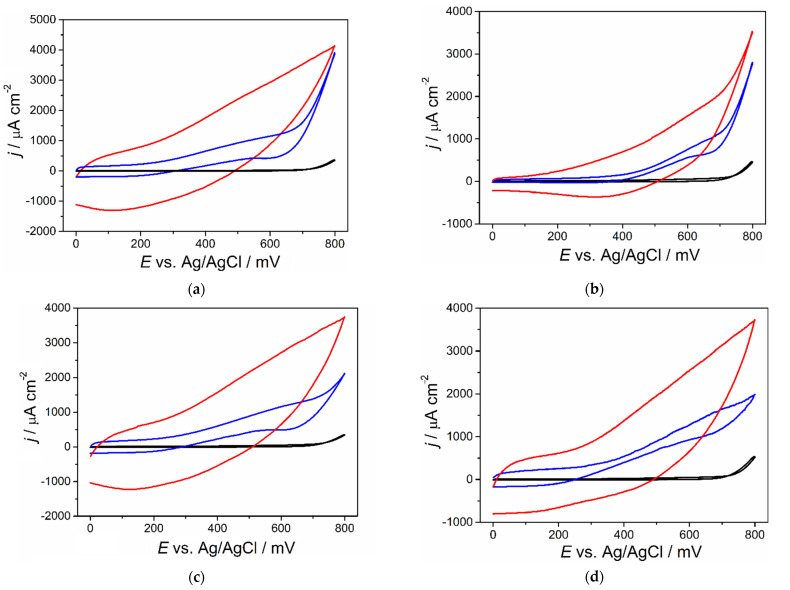
Cyclic voltammograms of 1 mmol L^−1^ saccharides in 0.1 mol L^−1^ NaOH using bare SPCE (black line), CuFE (blue line) and pCuFE (red line) and scan rate 50 mV s^−1^: (**a**) glucose; (**b**) sucrose; (**c**) fructose; (**d**) galactose. Each experiment was performed on a newly prepared sensor.

The electrochemical properties of pCuFEs in the detection of saccharides were investigated using cyclic voltammetry and compared to a non-porous CuFE and bare SPCE. The general mechanism of direct electrooxidation of saccharides at copper-based electrodes includes adsorption and deprotonation of the organic molecule on the electrode surface and subsequent oxidation with Cu(II)/(III) redox couple in copper oxides formed at positive potentials in alkaline media (Scheme 1) [28,31]. All current responses were recalculated to the corresponding current densities with respect to the geometric surface of the used electrodes for better comparison. Cyclic voltammograms recorded for 1 mmol L^−1^ saccharides in 0.1 mol L^−1^ NaOH using a scan rate of 50 mV s^−1^ (Figure 2) showed an intensive current response for electrooxidation of the compounds at the pCuFE compared to that obtained at the non-porous CuFE. No response is seen at bare SPCE where saccharides are not apparently oxidized at all. The pH value of the supporting electrolyte plays an important role in the electrocatalytic process. It was found that oxidation current response practically disappears in solutions with a pH below 10. The most intensive oxidation currents of saccharides were observed in 0.1 mol L^−1^ NaOH (pH 13), which was then thoroughly used in the following experiments.

It is generally known that porous electrodes exhibit several times higher sensitivity compared to non-porous electrodes in the electrochemical detection of compounds, which adsorb at the electrode surface [35]. The pCuFE possesses an advantage over the non-porous CuFE in providing more active sites in the same geometric dimensions for the adsorption of the saccharide, which can undergo a redox process afterward. It is evident from the cyclic voltammograms in Figure 2 that the electrooxidation of saccharides is highly pronounced at the pCuFE, which facilitates the adsorption of the analyte, resulting in much higher oxidation currents.

### 3.3. Analytical Performance of the pCuFE in the Electrochemical Detection of Saccharides

**Figure 3:**
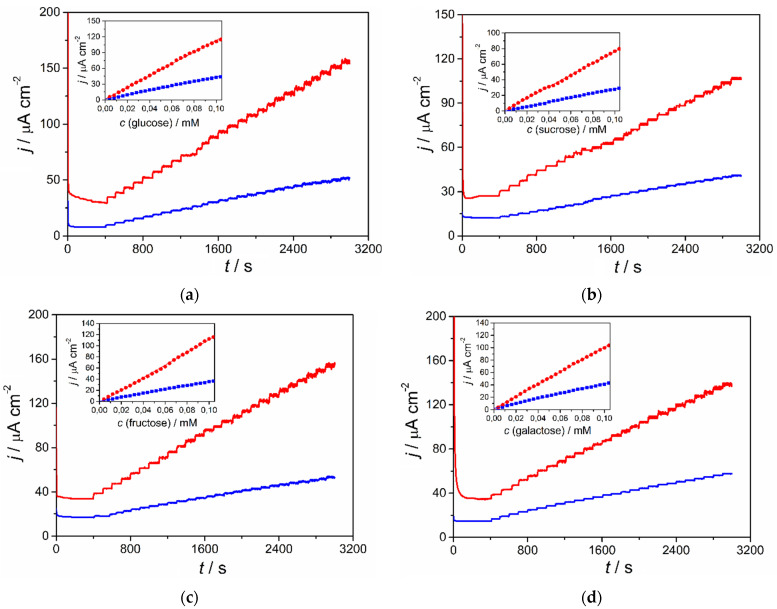
Hydrodynamic amperometry of saccharides within concentration range 4 × 10^−6^–1 × 10^−4^ mol L^−1^ saccharides in 0.1 mol L^−1^ NaOH at +0.6 V vs. Ag/AgCl using CuFE (blue line) and pCuFE (red line): (**a**) glucose; (**b**) sucrose; (**c**) fructose; (**d**) galactose. Each experiment was performed on a newly prepared sensor.

The analytical parameters of the pCuFE in the detection of saccharides were evaluated and compared to those of the non-porous CuFE using hydrodynamic amperometry in batch conditions. The detection potential of +0.6 V vs. Ag/AgCl was selected according to the cyclic voltammograms of saccharides recorded in 0.1 mol L^−1^ NaOH (Figure 2). At this potential, the electrocatalytic oxidation current is already on a high level and sufficiently far away from increasing background currents at more positive potentials. Figure 3 shows examples of the corresponding amperograms for all analyzed saccharides, obtained after successive additions of the compounds into the supporting electrolyte stirred with a Teflon^®^ bar at 600 rpm in a measuring vessel. The insets in Figure 3 display calibration lines for the concentration range 4 × 10^−6^–1 × 10^−4^ mol L^−1^ from current values evaluated 50 s after the addition.

It can be clearly seen that pCuFE exhibits much higher current changes upon the increase of the concentration of the analyte than non-porous CuFE. The signal stabilizes quickly at both copper film electrodes, and a larger noise is only visible at higher concentrations, presumably owing to the changes on the electrode surface, which is permanently exposed to positive potential during the prolonged time of the measurement. The sensitivities in saccharides detection, expressed as slopes of the corresponding calibration lines, were several times higher at pCuFE compared to those at CuFE: 2.7 times, 2.6 times, 3.2 times, and 2.5 times higher for glucose, sucrose, fructose, and galactose, respectively, for concentration range 4 × 10^−6^–1 × 10^−4^ mol L^−1^ (Table 1).

For the lower concentration range 2 × 10^−7^–1 × 10^−6^ mol L^−1^, the corresponding sensitivities were 1.8 times, 3.5 times, 7.5 times, and 2.4 times higher for glucose, sucrose, fructose, and galactose, respectively. The lower sensitivities obtained for the higher concentration range in comparison with those determined in the lower concentration range are probably a result of the ongoing saturation of the electrode surface with the analyte.

The limits of detection (LOD) for saccharide detection were calculated as three times the standard deviation of currents recorded for the lowest values of the linear range and measured using five individual sensors, divided by the slope averaged from five individual calibrations of those sensors in the concentration range 2 × 10^−7^–1 × 10^−6^ mol L^−1^. Such a method of LOD calculation considers the electroanalytical properties of several independently prepared sensors and thus will indicate the robustness of the pCuFEs prepared using colloidal crystal templating in real analyses. Obtained values of LODs are summarized in Table 1 together with the corresponding sensitivities for the lower and higher concentration range of saccharides. As it can be seen, the limits of detection are comparable to the lowest values in already published non-enzymatic copper-based sensors for glucose and are much lower for galactose.

As can be expected, most sugars will provide a current response, and therefore for samples containing a mixture of different saccharides, the obtained current response will correspond to their total amount. Herein, it is important to realize that already developed non-enzymatic sensors have faced the same problem. It is evident that the applicability of pCuFEs only depends on the appropriate selection of samples. For example, in clinical analysis, the literature reports up to 100-fold higher glucose concentration than galactose in plasma [36,37]. The content of other sugars, especially mannose and fructose, in human blood plasma and serum is an almost trace amount (tens-of-micromolar concentrations) [38,39]. The limit of UA in blood plasma for a healthy person is around 0.4 mmol L^−^^1^, whereas for glucose it is around 5 mmol L^−^^1^ [40,41].

### 3.4. Interference Study and Repeatability

Ascorbic (AA) and uric acid (UA) can be considered as predominant interfering species for the non-enzymatic detection of saccharides in clinical and food analysis. As evident from Table 1, other potential interferents (DA and APAP) are usually not the target of interest because they occur in low concentration levels, namely 0.2–2.0 nmol L^−1^ DA [42] in plasma or 50 μmol L^−1^ APAP in urine [43,44]. Regarding food samples, it is important to realize that sugars are one of the three main macronutrients in comparison with vitamins and minerals. It is known that the content of AA in fruit juices is up to 430 mg L^−1^, whereas juices or sweet drinks contain up to tens of grams per liter of saccharides [45]. Hence, the interference effect of AA and UA on current signals of saccharides was not necessary to investigate for comparable (1:1) and higher concentrations. Moreover, other organic species are oxidized very often from potentials +0.8 to 1.0 V in neutral or acidic media and are not contributing significantly to the amperometric signal at +0.6 V in a strongly alkaline environment. Such a combination of factors is very favorable for copper-based non-enzymatic sensors for their practical use.

It is generally known that the electroanalysis of AA and UA is based on irreversible oxidation, which was proven to be a diffusion-controlled process. This fact was confirmed during the interference study when both AA and UA were analyzed together with saccharides using hydrodynamic amperometry. Both interferants exhibited almost the same current densities during amperometric detection regardless of the working electrode. The enlarged porous surface of the pCuFE in comparison with the CuFE did not cause increased current densities for AA and UA oxidation, which is in concordance with [35].

**Figure 4:**
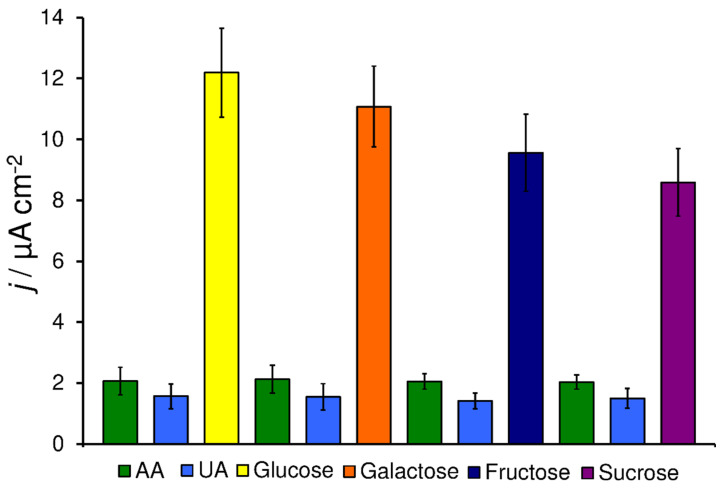
Repetitive sequential analyses of 6 × 10^−6^ mol L^−1^ AA and UA and 6 × 10^−5^ mol L^−1^ saccharides at one pCuFE. Hydrodynamic amperometry in 0.1 mol L^−1^ NaOH at *E*_d_ = +0.6 V vs. Ag/AgCl, errors bars show standard deviation of the measurement (*n* = 8).

The advantage of the use of pCuFE over CuFE for the detection of saccharides in the presence of AA or UA was demonstrated in the following experiments (Figure 4). For sequential injections of 1 × 10^−5^ mol L^−1^ AA and UA and 1 × 10^−4^ mol L^−1^ saccharides (ratio 1:10), the measured currents for AA constituted only 10–14% of the response of saccharides at pCuFE, whereas it was 14–24% when CuFE was used. The values within 6–11% and 12–25% for pCuFE and CuFE, respectively, were obtained in the case of UA injections. The porous surface of pCuFE improves the sensitivity of saccharides detection resulting in a smaller overall contribution from AA and UA.

The repeatability of amperometric detection of saccharides in the presence of AA and UA at the pCuFE was determined for repetitive analyses of 6 × 10^−6^ mol L^−1^ AA and UA and 6 × 10^−5^ mol L^−1^ saccharides (ratio 1:10). The AA, UA, and saccharides were sequentially analyzed eight times at one pCuFE electrode, and average currents from eight measurements together with the corresponding relative standard deviations (RSD) were plotted in Figure 4. After each addition of compounds, the current was left to stabilize for 200 s prior to the next addition, so the total time of all experiments surveyed in Figure 4 was over 5 h using only one sensor. The resulting RSD values for repetitive measurements were around 12% for all analyzed saccharides. It was confirmed experimentally that the storage stability of the pCuFEs is several months after preparation when stored in a dark and cold place.

## 4. Conclusions

The macroporous copper film electrode was successfully prepared with full coverage of the porous layer on the surface of the screen-printed carbon electrode using colloidal crystal templating and an optimized procedure for copper electrodeposition. The enlarged electrode surface enables more sensitive electrochemical detection of species, which adsorb firstly at the electrode surface prior to the electrochemical redox transformation, which is typical for electroanalysis of various saccharides. The contribution from interfering species is significantly reduced in the case of compounds with diffusion-controlled redox activity. We have demonstrated that it is feasible to detect saccharides at submicromolar concentrations with the aid of the porous copper film screen-printed carbon sensors. These sensors provide two linear dependencies for all examined saccharides, 0.2–1 and 4–100 µmol L^−1^. The procedure for the porous layer preparation is very simple, low cost, and demands only controlled conditions for the proper formation of the colloidal crystal template. With such an approach, porous structures of metals with defined pore dimensions can be easily prepared, and their properties can be tuned for particular electroanalytical applications. Due to their relatively high mechanical stability (more than half a year depending on storage conditions), it is planned to utilize these porous copper film screen-printed electrodes as amperometric detectors in flow injection analysis, allowing quick analysis of saccharides in many samples.

## Figures and Tables

**Table 1:** Overview of non-enzymatic electrochemical detection of saccharides using porous copper-based electrodes.

Sensor	Saccharide	Sensitivity (µA L mmol^−1^ cm^−2^)	Linear Range (µmol L^−1^)	LOD (µmol L^−1^)	*E*_d_ (V)	Medium	Selectivity	Ref.
CuF	Glucose	2570, 1810	2–80, 100–5000	0.98	+0.35	0.10 mol L^−1^ NaOH	AA, UA, DA, APAP	[6]
CuONHs	Glucose	5984	0.2–2000	0.3	+0.50	0.10 mol L^−1^ NaOH	AA, UA, Suc, Mal, Lac	[8]
Cu(OH)_2_/PGF	Glucose	3360	1.2–6000	1.2	+0.60	1.0 mmol L^−1^ NaOH	AA, UA, DA, Frc, Lac	[9]
CuONWA/CF	Glucose	32,330	0.1–500	0.02	+0.50	0.10 mol L^−1^ NaOH	AA, UA, DA, Frc, Lac, Mal, Sor	[10]
CuF	Glucose	5850	1–500	0.5	+0.55	0.10 mol L^−1^ NaOH	AA, DA, UA	[12]
CuO/PrGO	Glucose	33	1–6000	0.05	+0.55	0.10 mol L^−1^ NaOH		[18]
pCuO/Nafion^®^	Glucose	11	2–350	1.3	+0.40	0.10 mol L^−1^ NaOH	AA, UA	[19]
pCuOF	Glucose	2900	1–2500	0.14	+0.65 *	0.10 mol L^−1^ NaOH	AA, UA	[21]
Cu–Cu_2_ONN	Glucose	124	10–5500	0.05	+0.60	0.10 mol L^−1^ NaOH	AA, DA, UA	[22]
CuFME	Glucose	1790	180–3470	9.3	+0.50	0.10 mol L^−1^ NaOH		[23]
CuFME	Galactose	570	180–3470	29.4	+0.50	0.10 mol L^−1^ NaOH		[23]
CuFME	Lactose	640	180–3470	26	+0.50	0.10 mol L^−1^ NaOH		[23]
pCu_2_OM	Glucose	71	up to 500	0.8	+0.61	0.10 mol L^−1^ NaOH		[24]
RGOs-Cu_2_O	Glucose	185	10–6000	0.05	+0.60 **	0.05 mmol L^−1^ KOH	AA, DA, UA	[25]
CuO-NWs/CF	Glucose	2217	1–18,800	0.3	+0.35	0.10 mol L^−1^ NaOH	AA, UA, DA	[26]
pCuFE	Glucose	34, 11	0.2–1, 4–100	0.1	+0.60	0.10 mol L^−1^ NaOH	AA, UA	[This work]
pCuFE	Fructose	50, 11	0.2–1, 4–100	0.03	+0.60	0.10 mol L^−1^ NaOH	AA, UA	[This work]
pCuFE	Sucrose	54, 8	0.2–1, 4–100	0.05	+0.60	0.10 mol L^−1^ NaOH	AA, UA	[This work]
pCuFE	Galactose	53, 10	0.2–1, 4–100	0.05	+0.60	0.10 mol L^−1^ NaOH	AA, UA	[This work]

Notes: AA; ascorbic acid, APAP; acetaminophen, CuF; copper foam, CuFME; cuprous foam electrode, Cu(OH)_2_/PGF; copper hydroxide nanorods decorated porous graphene foam electrode, CuONHs; CuO nanothorns, CuONWA/CF; three-dimensional copper foam supported CuO nanowire arrays, CuO-NWs/CF; CuO nanowires on three-dimensional porous copper foam, CuO/PrGO; copper oxide supported on three-dimensional porous reduced graphene oxide, pCu_2_OM; porous Cu_2_O microcubes, Cu–Cu_2_ONN; Cu–Cu_2_O nanoporous nanoparticles, DA; dopamine, Frc; fructose, Lac; Lactose, Mal; maltose, pCu_2_O/Nafion^®^; porous cuprous oxide to a Nafion film, pCuOF; porous CuO electrode fabricated by hydrogen bubble evolution, RGOs-Cu_2_O; monodisperse porous Cu_2_O nanospheres on reduced graphene oxide, Sor; sorbitol, Suc; sucrose, UA; uric acid. * vs. Hg/HgO reference; ** vs. saturated calomel electrode reference.

## Data Availability

The data presented in this study are available on request from the corresponding author.
